# Cerebral Infarction: Epidemiology, Classification, Mechanisms, Diagnosis, and Management

**DOI:** 10.1002/mco2.70704

**Published:** 2026-04-01

**Authors:** Meibiao Zhang, Jianxun Hou, Jia Kuang, Lei Hao, Zhao Yang

**Affiliations:** ^1^ Department of Neurology The Affiliated Yongchuan Hospital of Chongqing Medical University Chongqing China

**Keywords:** biomarkers, ischemic stroke, multiomics, neuroimaging, pathophysiology, precision medicine

## Abstract

Cerebral infarction, the pathological basis of ischemic stroke, remains a leading cause of mortality and long‐term disability worldwide. Its clinical heterogeneity reflects the complex interplay among vascular pathology, metabolic failure, immune responses, and genetic susceptibility, posing persistent challenges to effective risk stratification and individualized therapy. This review provides an integrative overview of cerebral infarction from a precision medicine perspective, synthesizing advances across epidemiology, pathophysiology, diagnostics, and therapeutics to bridge the gap between mechanistic insight and clinical outcomes. We first summarize global epidemiological trends, highlighting persistent disparities and the shift toward understanding modifiable and emerging risk factors. We then critically examine the evolution of stroke classification from traditional systems (e.g., Trial of Org 10172 in Acute Stroke Treatment [TOAST]) toward phenotype‐driven and molecularly informed frameworks. The core of the review delves into key pathophysiological mechanisms—including neurovascular unit dysfunction, energy metabolism disturbance, and regulated cell death pathways such as ferroptosis—and their implications for targeted intervention. We further appraise contemporary diagnostic advances, encompassing multimodal imaging, circulating biomarkers, and artificial intelligence‐assisted tools, alongside current treatment strategies like reperfusion therapy and emerging neuroprotective approaches. Finally, we discuss how multiomics technologies and data‐driven models are redefining stroke subtyping and guiding individualized management. By employing a mechanism–phenotype–decision framework, this review offers a coherent synthesis of the field, providing a roadmap to support the transition from empirical care toward precision‐oriented management in cerebral infarction.

## Graphical abstract

Within minutes after ischemia onset, deprivation of oxygen and glucose leads to rapid ATP depletion, resulting in failure of Na^+^/K^+^‐ATPase activity and membrane depolarization. This ionic imbalance promotes uncontrolled synaptic release of glutamate and excessive activation of postsynaptic NMDA and AMPA receptors. Subsequent massive influx of calcium and sodium ions triggers excitotoxic neuronal injury, representing one of the earliest and most potent molecular drivers of ischemic cell death.

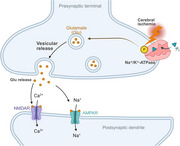

## 1 Introduction

Cerebral infarction represents the core pathological substrate of ischemic stroke and results from an abrupt or sustained reduction in cerebral blood flow (CBF), leading to irreversible focal brain injury. According to Global Burden of Disease (GBD) analyses, stroke remains one of the leading causes of mortality and long‐term disability worldwide, with ischemic stroke accounting for approximately 70–80% of all stroke cases [1, 2, 3]. Although age‐standardized incidence and mortality rates have declined in several high‐income countries (HICs), the absolute number of patients affected by cerebral infarction continues to rise, largely driven by population aging and the cumulative burden of vascular risk factors [4, 5, 6]. Since the latter half of the twentieth century, conceptual frameworks of cerebral infarction have evolved substantially—from a simplistic model of arterial occlusion to the ischemic cascade and more recently to integrative paradigms emphasizing the neurovascular unit (NVU) and systemic regulatory networks [7, 8]. These shifts have profoundly shaped both experimental research and clinical practice.

Over the past decade, major advances have been achieved in epidemiological surveillance, etiological classification, neuroimaging, and acute management of cerebral infarction. The widespread adoption of multimodal computed tomography (CT) and magnetic resonance imaging (MRI) has enabled refined assessment of ischemic core and penumbral tissue, supporting extended therapeutic windows for endovascular thrombectomy [9, 10, 11]. In parallel, growing evidence has elucidated key molecular and cellular mechanisms underlying ischemic injury, including neuroinflammation, mitochondrial dysfunction, blood–brain barrier (BBB) disruption, and regulated cell death pathways such as ferroptosis [12, 13, 14]. Despite these advances, substantial gaps remain between technological progress and real‐world clinical outcomes. A large proportion of patients remain ineligible for reperfusion therapies due to temporal or anatomical constraints, and even successful recanalization is frequently followed by no‐reflow phenomena or secondary injury [15, 16]. Moreover, conventional etiological classifications, including TOAST and embolic stroke of undetermined source (ESUS), often fail to capture the coexistence of multiple pathogenic mechanisms, thereby limiting accurate risk stratification and individualized therapeutic decision‐making [17, 18].

These unresolved challenges highlight a fundamental issue: cerebral infarction is not a single disease entity, but rather a highly heterogeneous clinical syndrome driven by complex interactions among vascular pathology, metabolic disturbances, immune responses, and genetic susceptibility. Existing reviews tend to focus on isolated dimensions, such as imaging, acute intervention, or molecular mechanisms—without providing a comprehensive, cross‐level synthesis. Meanwhile, rapid developments in multiomics technologies, artificial intelligence (AI), and digital medicine are accelerating a paradigm shift in stroke research, from population‐based averages toward mechanism‐oriented, individualized management strategies [19, 20]. In this context, a systematic and integrative reassessment of cerebral infarction across epidemiological, mechanistic, diagnostic, and therapeutic domains is both timely and necessary to support the transition toward precision stroke medicine.

Accordingly, this review is structured to progress sequentially from epidemiology, outlining the global burden and population disparities of cerebral infarction, to mechanisms, with a focus on NVU dysfunction, energy metabolism failure, and regulated cell death. Advances in diagnosis, including neuroimaging, circulating biomarkers, and AI‐assisted tools, are then summarized, followed by an appraisal of contemporary treatment strategies encompassing reperfusion therapy, secondary prevention, and emerging interventions. Finally, the review addresses precision medicine, discussing how multiomics integration and intelligent technologies may redefine stroke subtyping and individualized care. The distinctive value of this review lies in its mechanism–phenotype–decision framework, which bridges basic science and clinical practice to provide a coherent roadmap for precision‐oriented management of cerebral infarction.

## 2 Epidemiology of Cerebral Infarction

Cerebral infarction, accounting for approximately 70–80% of all strokes worldwide, remains a dominant contributor to global mortality, long‐term disability, and healthcare expenditure. Over the past three decades, advances in vascular risk factor control and acute stroke care have led to declining age‐standardized incidence and mortality rates in several HICs. Nevertheless, the absolute burden of cerebral infarction continues to rise globally, driven by population aging, demographic expansion, and the uneven distribution of preventive and therapeutic resources. Contemporary epidemiological research has shifted from merely describing incidence and mortality toward understanding regional disparities, evolving risk profiles, and emerging environmental and genetic determinants. A comprehensive appraisal of these trends is essential to inform targeted prevention strategies and health policy planning.

### 2.1 Global Incidence, Mortality, and Disability Burden

According to the GBD 2021 study, stroke remains the second leading cause of death and the third leading cause of death and disability combined worldwide [1, 2, 3]. In 2019, there were an estimated 12.2 million incident strokes, over 101 million stroke survivors, and approximately 6.55 million stroke‐related deaths globally, with ischemic stroke representing the majority of cases [2, 3]. Disability‐adjusted life‐years (DALYs) attributable to stroke exceeded 143 million, reflecting a >30% increase since 1990 [1].

Despite modest declines in age‐standardized incidence and mortality rates, the absolute number of cerebral infarction cases continues to rise, particularly in aging populations [4, 5]. Modeling studies project that without intensified prevention efforts, global stroke deaths could exceed 9 million annually by 2030 [6]. Importantly, cerebral infarction disproportionately contributes to long‐term disability, with nearly half of survivors experiencing persistent motor, cognitive, or emotional impairment [1].

### 2.2 Geographic and Socioeconomic Disparities

A striking feature of cerebral infarction epidemiology is its uneven global distribution. Over 70% of stroke‐related deaths and approximately 77% of stroke DALYs occur in low‐ and middle‐income countries (LMICs) [3, 8]. While HICs have achieved substantial reductions in stroke mortality through effective hypertension control, smoking cessation, and organized stroke care systems, LMICs have experienced a >100% increase in stroke incidence over the past four decades [7, 10].

Limited access to primary prevention, delayed hospital presentation, scarcity of thrombolysis and thrombectomy services, and underdeveloped rehabilitation infrastructure contribute to worse outcomes in resource‐limited settings [9, 11]. Even within HICs, socioeconomic gradients persist, with individuals from disadvantaged communities experiencing higher incidence, poorer access to acute care, and increased poststroke disability [3].

### 2.3 Age, Sex, and Ethnic Differences

Age remains the most powerful nonmodifiable risk factor for cerebral infarction. Incidence increases exponentially after the age of 55 years, with individuals over 65 years of age accounting for more than two‐thirds of all ischemic strokes [14]. Alarmingly, recent epidemiological studies have documented a rising incidence of cerebral infarction among younger adults (<50 years), likely reflecting increasing prevalence of obesity, diabetes, hypertension, and substance use [12, 13].

Sex‐related differences are also pronounced. Men exhibit higher age‐adjusted incidence rates in early and mid‐adulthood, whereas women carry a higher lifetime risk and experience greater stroke severity, disability, and mortality [15, 16]. These differences are partially explained by longer life expectancy and sex‐specific risk factors, including pregnancy‐related complications, hormonal influences, and a higher prevalence of atrial fibrillation in older women [17].

Racial and ethnic disparities further compound the global burden. Black, Hispanic, and South Asian populations consistently demonstrate higher incidence, earlier onset, and worse functional outcomes compared with White populations [18, 19, 20]. These disparities reflect complex interactions between socioeconomic determinants, healthcare access, comorbid risk factors, and potential genetic susceptibility.

### 2.4 Modifiable Risk Factors and Population‐Attributable Risk

**FIGURE 1:**
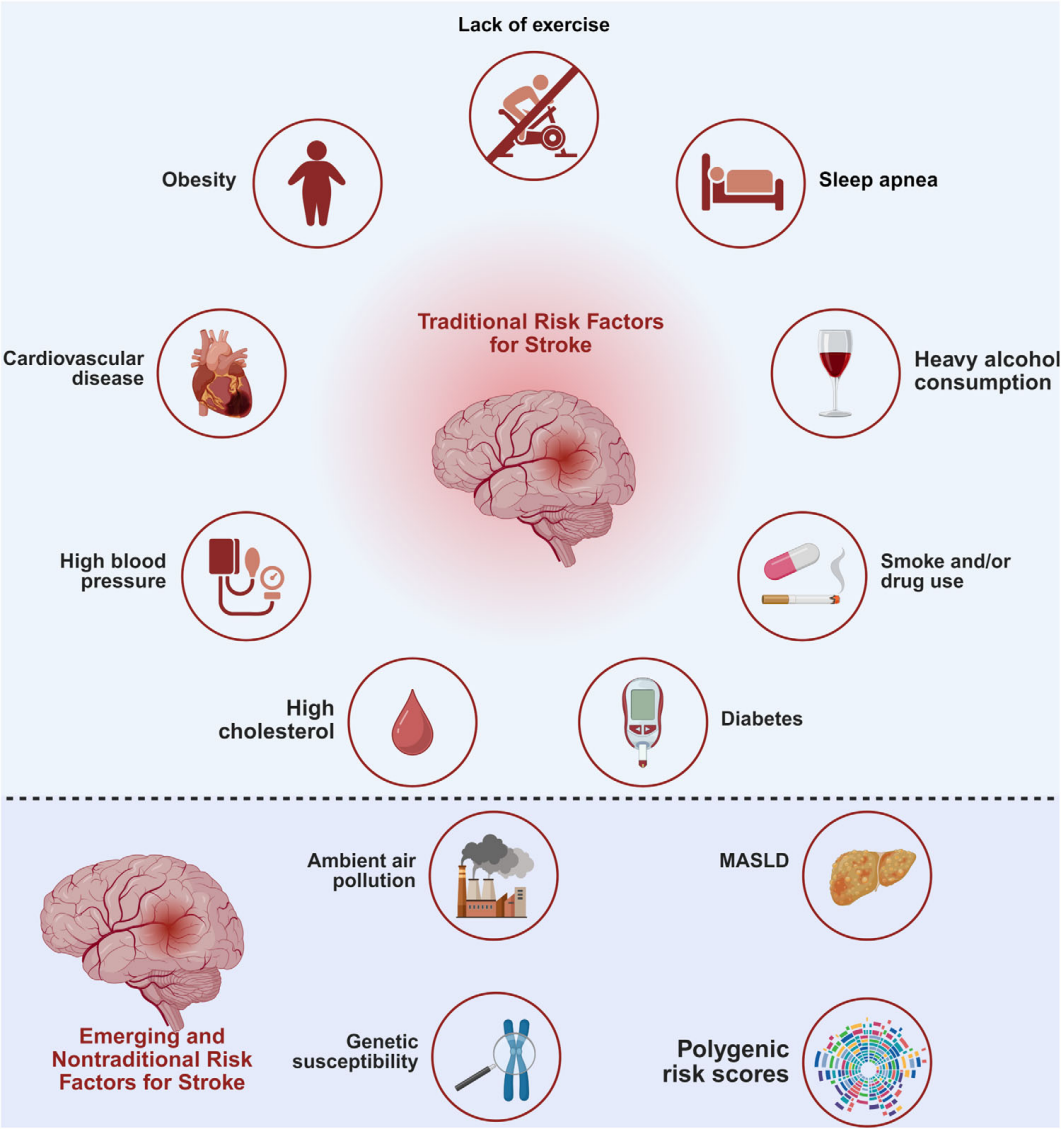
Traditional and emerging risk factors for cerebral infarction. The figure contrasts established modifiable risk factors, which account for the majority of population‐attributable risk, with a growing list of nontraditional contributors. Established factors (upper panel) are led by hypertension, followed by dyslipidemia, diabetes, smoking, and physical inactivity. Emerging factors (lower panel), including air pollution, metabolic dysfunction‐associated steatotic liver disease (MASLD), and genetic susceptibility, are thought to promote infarction through convergent proinflammatory, endothelial, and prothrombotic mechanisms.

Modifiable risk factors account for over 90% of the population‐attributable risk of cerebral infarction worldwide [15]. Hypertension remains the single most important contributor, responsible for nearly half of all ischemic strokes [21]. Additional major risk factors include dyslipidemia, diabetes mellitus, smoking, abdominal obesity, physical inactivity, unhealthy diet, atrial fibrillation, and excessive alcohol consumption [15, 22], as illustrated in the upper half of Figure 1.

Large population‐based studies have demonstrated that aggressive risk factor modification—particularly blood pressure reduction and statin therapy—can substantially lower both primary and recurrent stroke risk [23, 24]. However, adherence to preventive strategies remains suboptimal, especially in socioeconomically disadvantaged populations, limiting their real‐world effectiveness [25].

### 2.5 Emerging and Nontraditional Risk Factors

Beyond traditional vascular risks, emerging contributors to cerebral infarction have gained increasing attention, as shown in the lower half of Figure 1. Ambient air pollution, particularly fine particulate matter (PM2.5), is now recognized as a major global stroke risk factor, accounting for over one million stroke‐related deaths annually [26, 27]. Mechanistically, PM2.5 exposure promotes systemic inflammation, endothelial dysfunction, and prothrombotic states [28].

Metabolic dysfunction‐associated steatotic liver disease (MASLD) has also been linked to increased ischemic stroke risk independent of traditional metabolic factors, likely through chronic inflammation and altered coagulation pathways [29, 30, 31]. In parallel, large‐scale Genome‐Wide Association Studies (GWAS) have identified hundreds of genetic loci associated with ischemic stroke and its subtypes, enabling the development of polygenic risk scores to stratify individual susceptibility [32, 33]. While clinical implementation remains limited, these tools hold promise for targeted prevention in high‐risk populations.

### 2.6 Conclusion

In summary, cerebral infarction remains a leading cause of death and disability worldwide, with a growing absolute burden despite improvements in age‐standardized rates. Profound geographic, socioeconomic, age‐, sex‐, and ethnicity‐related disparities persist, underscoring the need for context‐specific prevention and care strategies. While traditional vascular risk factors continue to dominate, emerging environmental, metabolic, and genetic contributors are reshaping the epidemiological landscape. A nuanced understanding of these trends is essential for informing public health policy, optimizing resource allocation, and advancing toward precision prevention strategies aimed at reducing the global impact of cerebral infarction.

## 3 Classification of Cerebral Infarction

Accurate classification of cerebral infarction is fundamental to etiological diagnosis, secondary prevention, and the rational design of clinical trials. Traditional classification systems, while clinically pragmatic, increasingly struggle to capture the biological heterogeneity underlying ischemic stroke. Over the past decade, advances in neuroimaging, cardiac diagnostics, and molecular profiling have catalyzed a paradigm shift from symptom‐based and single‐cause frameworks toward multidimensional, mechanism‐oriented phenotyping. This section critically reviews classical and contemporary classification systems, highlights their respective strengths and limitations, and discusses emerging precision‐based approaches that aim to redefine stroke subtyping in the modern era.

### 3.1 Classical Clinical and Syndromic Classifications

Early classification systems were primarily syndromic, relying on clinical presentation and anatomical localization. The Oxfordshire Community Stroke Project (OCSP) classification categorizes infarctions into total anterior circulation infarct, partial anterior circulation infarct, lacunar infarct, and posterior circulation infarct. Despite its simplicity and prognostic utility, OCSP lacks etiological specificity and offers limited guidance for targeted secondary prevention [34, 35, 36]. Its continued use today is largely confined to epidemiological studies and early bedside stratification rather than mechanistic inference.

### 3.2 Etiology‐Oriented Systems: TOAST and Its Refinements

The TOAST classification remains the most widely applied etiological framework, categorizing ischemic stroke into large‐artery atherosclerosis, cardioembolism, small‐vessel occlusion, other determined causes, and undetermined etiology [37]. Although TOAST has demonstrated reasonable inter‐rater reliability, contemporary studies consistently report that 25–30% of patients are labeled as cryptogenic, underscoring diagnostic insufficiency rather than true etiological ambiguity [37, 38]. Moreover, TOAST enforces a single‐cause paradigm, inadequately reflecting the frequent coexistence of multiple pathogenic substrates—such as atrial fibrillation with concomitant atherosclerosis—in aging populations [39].

To address these limitations, modified TOAST‐based algorithms incorporating prolonged cardiac monitoring and advanced vascular imaging have modestly reduced the cryptogenic fraction, yet substantial uncertainty persists [40]. A summary comparison of TOAST categories and their characteristics is provided in Table 1.

**TABLE 1:** Comparison of TOAST and ASCO/ASCOD classification systems for ischemic stroke.

System	Category	Definition/description	Key features/notes
TOAST [37, 38, 39, 40]	Large‐artery atherosclerosis	Stroke due to ≥50% stenosis or occlusion of major extracranial or intracranial arteries	Single‐cause assignment; widely used; simple to apply
	Cardioembolism	Stroke attributable to cardiac sources (e.g., atrial fibrillation, recent myocardial infarction)	
	Small‐vessel occlusion	Lacunar infarcts caused by small‐vessel disease	
	Other determined causes	Rare etiologies (e.g., arterial dissection, vasculitis)	
	Undetermined	No clear cause after standard evaluation	Cryptogenic cases ∼25–30%; limited reflection of multifactorial etiology
ASCO [41]	Atherosclerosis (A)	Presence of large‐artery atherosclerotic disease	Graded, phenotype‐based; multiple potential etiologies can be scored
	Small‐vessel disease (S)	Evidence of small‐vessel pathology	
	Cardiac pathology (C)	Cardiac conditions contributing to stroke	
	Other causes (O)	Other potential etiologies	Improves recognition of multifactorial stroke; complexity may limit routine clinical use
ASCOD [42, 43]	Atherosclerosis (A)	Distinguishes presence from causal relevance	Further refines ASCO; allows scoring of multiple etiologies; improves mechanistic plausibility
	Small‐vessel disease (S)		
	Cardiac pathology (C)		
	Other causes (O)		
	Dissection (D)	Added category in ASCOD	Captures additional rare but relevant causes

Abbreviations: ASCO, atherosclerosis, small‐vessel disease, cardiac pathology, other causes; ASCOD, ASCO + Dissection; SVD, small‐vessel disease; TOAST, Trial of Org 10172 in Acute Stroke Treatment.

### 3.3 Phenotypic Classification: The ASCO and ASCOD Systems

The ASCO classification (atherosclerosis, small‐vessel disease, cardiac pathology, other causes) introduced a graded, phenotype‐based model that allows multiple potential etiologies to be scored simultaneously [41]. Its updated version, ASCOD, further distinguishes disease presence from causal relevance, improving granularity and mechanistic plausibility [42]. These systems acknowledge stroke as a multifactorial disorder rather than a unidimensional event, enhancing suitability for research and precision prevention. However, their complexity limits routine clinical adoption, and standardized thresholds for causal attribution remain debated [43]. Table 1 also illustrates the corresponding categories and features of ASCO and ASCOD, highlighting differences with the TOAST framework.

### 3.4 Imaging‐Based and Tissue‐Oriented Classification

The widespread availability of diffusion‐weighted imaging (DWI) and perfusion techniques has enabled tissue‐based classification centered on infarct pattern, size, and temporal evolution. Distinct lesion topographies—such as multiple acute infarcts across vascular territories or cortical–subcortical borderzone patterns—provide powerful clues to underlying mechanisms, particularly cardioembolism and hemodynamic failure [44, 45]. High‐resolution vessel wall imaging (VWI) has further refined etiological inference by differentiating plaque rupture, intraplaque hemorrhage, dissection, and nonatherosclerotic vasculopathies [46, 47]. Such imaging phenotypes increasingly complement, and in some contexts supersede, purely clinical classifications.

### 3.5 Cryptogenic Stroke and ESUS: Conceptual Advances and Controversies

The construct of ESUS was proposed to define nonlacunar infarcts without major cardioembolic sources or significant arterial stenosis [48]. While conceptually appealing, large randomized trials testing empiric anticoagulation in ESUS failed to demonstrate benefit over antiplatelet therapy, revealing profound biological heterogeneity within this category [36, 49]. Subsequent analyses suggest that ESUS encompasses multiple distinct mechanisms, including occult atrial cardiopathy, nonstenotic vulnerable plaques, and cancer‐associated coagulopathy [37, 44, 50].

### 3.6 Toward Precision Stroke Subtyping

Contemporary classification increasingly emphasizes mechanism‐informed endophenotypes. The concept of atrial cardiopathy—defined by structural, electrical, or biomarker abnormalities independent of atrial fibrillation—has emerged as a promising subtype with therapeutic implications [42, 43]. Parallel advances in plasma biomarkers, genomics, and transcriptomics have identified molecular signatures associated with specific stroke mechanisms, such as inflammatory atherosclerosis or hypercoagulable states [39, 40, 45]. Integration of these data with advanced imaging through machine‐learning (ML) frameworks is actively being explored to generate reproducible, clinically actionable stroke subtypes [36, 41, 50].

### 3.7 Conclusion

The classification of cerebral infarction is undergoing a decisive transformation. While traditional systems such as TOAST and OCSP retain historical and pragmatic value, they are increasingly insufficient to capture the mechanistic complexity of modern stroke populations. Emerging phenotype‐driven, imaging‐based, and molecularly informed frameworks offer a more nuanced and biologically coherent approach, aligning classification with therapeutic decision‐making and trial design. Ultimately, the future of stroke classification lies in integrative models that synthesize clinical features, tissue signatures, cardiac phenotypes, and omics data‐laying the foundation for truly precision‐guided prevention and treatment.

## 4 Pathophysiological and Molecular Mechanisms of Cerebral Infarction

Cerebral infarction is no longer conceptualized as a linear ischemic cascade culminating in inevitable neuronal necrosis, but rather as a highly dynamic, spatiotemporally regulated systems‐level failure involving neurons, glial cells, vascular elements, immune components, and systemic metabolic signals [51, 52, 53]. Advances in single‐cell sequencing, high‐resolution in vivo imaging, and multiomics profiling over the past decade have fundamentally reshaped our understanding of ischemic brain injury, revealing profound heterogeneity across cell types, brain regions, and temporal stages of stroke evolution [54, 55, 56]. Within this framework, the ischemic core, peri‐infarct penumbra, and remote yet functionally connected brain regions represent distinct but interacting microenvironmental niches, each governed by partially overlapping injury programs.

Importantly, the mechanisms determining tissue fate after arterial occlusion are neither uniform nor deterministic. Instead, they are shaped by premorbid vascular integrity, collateral circulation efficiency, immune priming status, metabolic reserve, and genetic susceptibility [57, 58]. These variables modulate not only infarct size but also the dominant molecular pathways activated in individual patients. Such mechanistic heterogeneity provides a compelling biological rationale for the transition toward precision stroke medicine, wherein therapeutic strategies are guided by prevailing injury mechanisms rather than applied indiscriminately. In this section, we synthesize contemporary evidence on the vascular, cellular, metabolic, inflammatory, and regulated cell‐death processes underpinning cerebral infarction, with an emphasis on emerging concepts of translational relevance.

### 4.1 Vascular Occlusion, Hemodynamics, and Endothelial Failure

The initiating event in cerebral infarction is a critical reduction in regional CBF, typically below 18–20 mL/100 g/min, resulting in immediate disruption of oxidative phosphorylation and aerobic energy metabolism [59]. However, tissue outcome is not dictated solely by the anatomical site of arterial occlusion, but by the functional integrity of the cerebrovascular network as a whole, including collateral circulation, microvascular patency, and endothelial homeostasis [60]. Robust collateral flow can sustain penumbral viability for hours, whereas poor collateralization accelerates infarct core expansion despite timely recanalization.

Atherosclerotic plaque rupture remains a dominant cause of large‐vessel cerebral infarction. Vulnerable plaques are characterized by lipid‐rich necrotic cores, thin fibrous caps, macrophage infiltration, and intraplaque neovascularization, features that predispose them to rupture and thrombosis [61]. Beyond mechanical obstruction, atherosclerosis represents a chronic inflammatory disorder that primes the downstream microvasculature for ischemic injury. Endothelial dysfunction—marked by impaired nitric oxide bioavailability, increased oxidative stress, and upregulation of adhesion molecules—both precedes and amplifies ischemic damage [62].

Recent studies have highlighted the critical role of endothelial glycocalyx degradation in stroke pathophysiology. Loss of this carbohydrate‐rich layer enhances leukocyte adhesion, platelet aggregation, and capillary plugging, contributing to the phenomenon of microvascular no‐reflow even after successful macrovascular recanalization [63, 64]. Failure of cerebral autoregulation further exacerbates ischemic vulnerability, rendering penumbral tissue exquisitely sensitive to systemic blood pressure fluctuations and therapeutic hypotension [65]. Moreover, ischemia‐induced pericyte constriction and death during reperfusion result in persistent capillary narrowing, reinforcing the concept that restoration of macrovascular patency alone is insufficient to guarantee tissue reperfusion [66].

### 4.2 Energy Failure, Excitotoxicity, and Ionic Dysregulation

**FIGURE 2:**
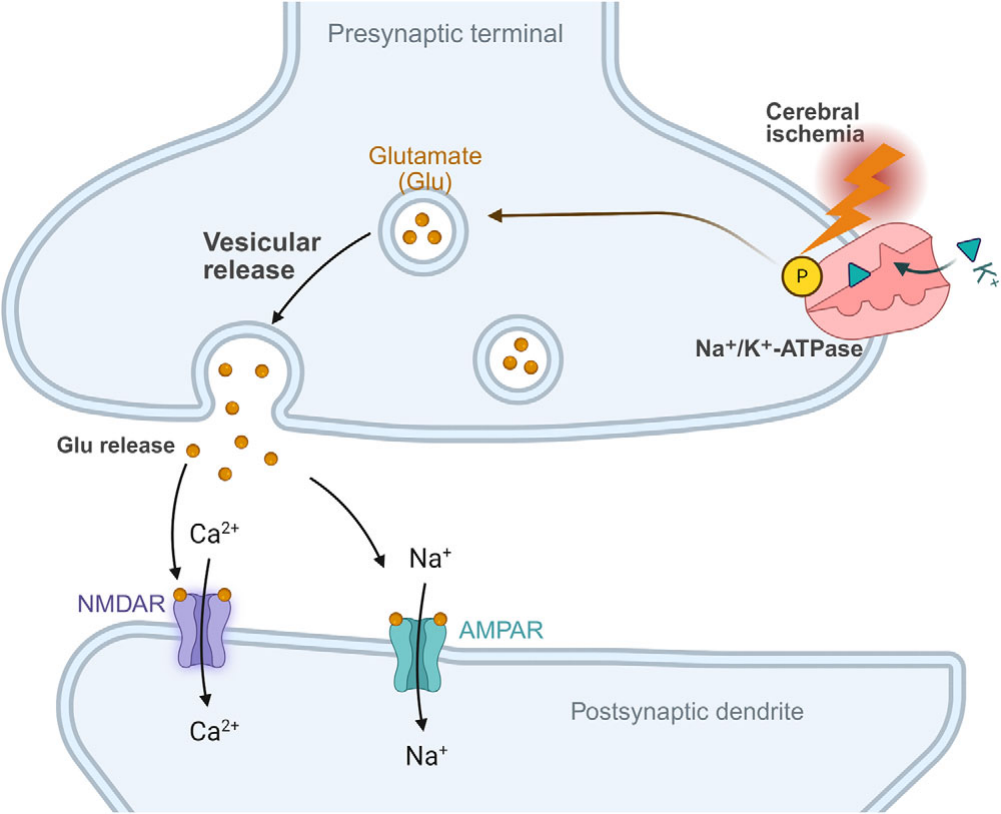
Ischemia‐induced energy failure and excitotoxic neuronal injury. This schematic illustrates the cascade of events from oxygen‐glucose deprivation to excitotoxicity. Following ischemia, rapid ATP depletion causes failure of the Na^+^/K^+^‐ATPase pump, leading to membrane depolarization and uncontrolled glutamate release. Overstimulation of postsynaptic NMDA and AMPA receptors triggers a massive influx of Ca^2^^+^ and Na^+^ ions, activating degradative enzymes and initiating excitotoxic cell death, one of the earliest drivers of ischemic injury.

Within minutes of ischemia onset, ATP depletion leads to failure of Na^+^/K^+^‐ATPase activity, membrane depolarization, and uncontrolled synaptic release of glutamate [67]. Excessive activation of NMDA and AMPA receptors permits massive influx of calcium and sodium ions, triggering excitotoxic neuronal injury [68]. As illustrated in Figure 2, this process represents one of the earliest and most potent drivers of ischemic cell death, particularly in the infarct core and inner penumbra.

Calcium overload functions as a central convergence point linking excitotoxicity to multiple downstream destructive pathways. Elevated intracellular calcium activates calpains, phospholipases, and endonucleases, leading to cytoskeletal breakdown, membrane degradation, and DNA fragmentation [69]. Sodium and chloride influx exacerbate cytotoxic edema, increasing intracranial pressure and further compromising microvascular perfusion [70]. Astrocytes initially exert protective effects by buffering extracellular glutamate and potassium; however, under sustained ischemic conditions, astrocytic energy failure leads to reversal of glutamate transporters and loss of homeostatic capacity, thereby amplifying excitotoxic stress.

### 4.3 Mitochondrial Dysfunction and Oxidative Stress

Mitochondria occupy a central position in ischemic injury, serving as both victims of metabolic collapse and active mediators of cell death. Calcium overload and oxidative stress disrupt mitochondrial membrane potential, precipitating opening of the mitochondrial permeability transition pore, collapse of ATP synthesis, and release of proapoptotic factors such as cytochrome *c* [71, 72]. These events commit neurons to irreversible injury, particularly when reperfusion is delayed.

Paradoxically, reperfusion itself intensifies injury through a burst of reactive oxygen and nitrogen species (ROS/RNS) generated by dysfunctional mitochondria, NADPH oxidases, and xanthine oxidase [73]. The resulting oxidative stress drives lipid peroxidation, protein nitration, and oxidative DNA damage, overwhelming endogenous antioxidant defenses and propagating secondary injury [74].

**FIGURE 3:**
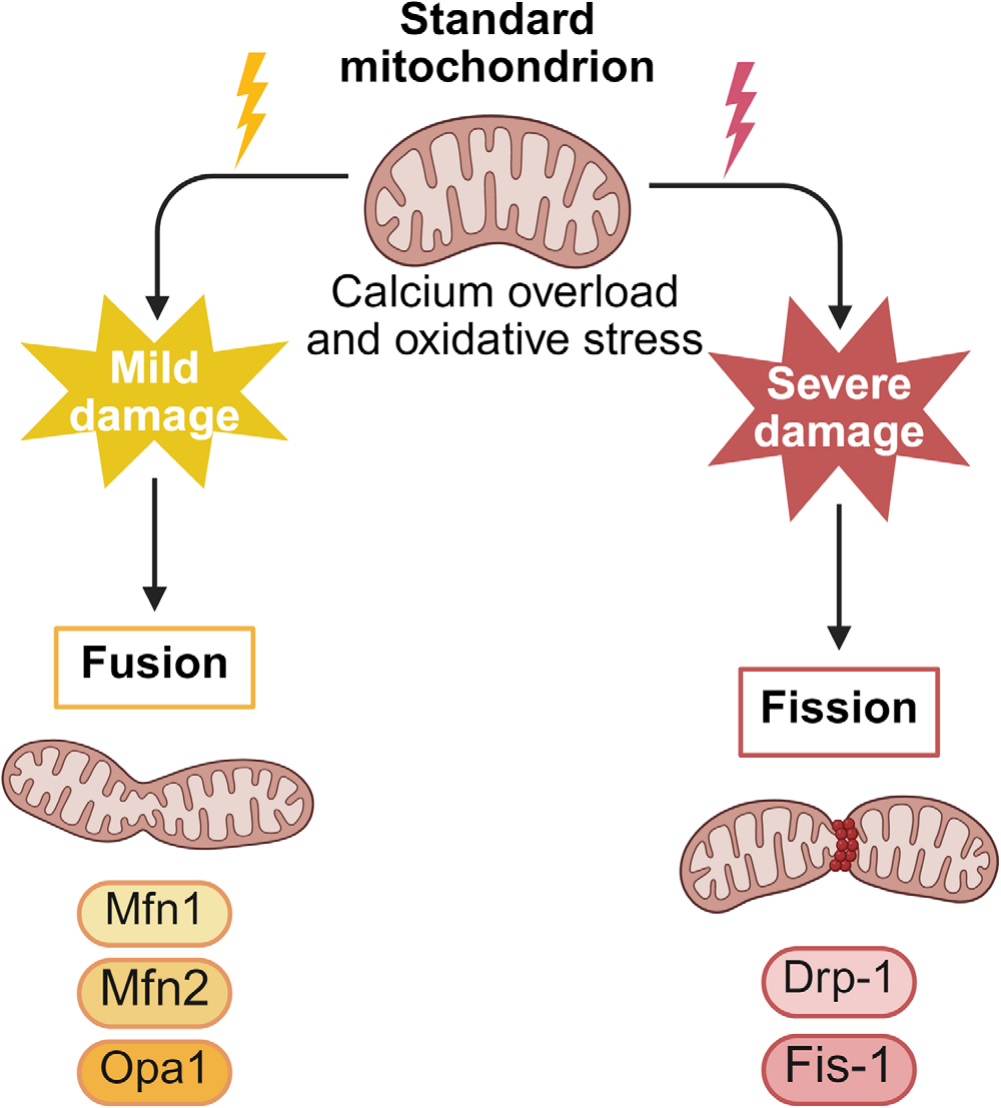
Mitochondrial dynamics imbalance in cerebral ischemia–reperfusion injury. The diagram depicts the shift from healthy mitochondrial dynamics to pathological fragmentation following ischemic stress. Ischemia–reperfusion induces calcium overload and ROS, which disrupt the fusion–fission balance. This is characterized by the downregulation of fusion proteins (Mfn1, Mfn2, OPA1) and upregulation of fission proteins (DRP1, Fis‐1), leading to excessive mitochondrial fragmentation. These dysfunctional mitochondria exhibit impaired ATP synthesis, increased ROS production, and release of proapoptotic factors, exacerbating neuronal injury.

Beyond bioenergetic failure, mitochondrial structural remodeling has emerged as a critical determinant of neuronal fate following ischemia–reperfusion injury. As illustrated in Figure 3, calcium overload and oxidative stress converge on mitochondrial dynamics regulatory machinery, leading to excessive fission and impaired fusion. Dysregulation of key proteins involved in fusion (Mfn1, Mfn2, and OPA1) and fission (DRP1 and Fis‐1) results in mitochondrial fragmentation, loss of cristae integrity, and further amplification of ROS production. In particular, excessive DRP1‐mediated fission coupled with compromised OPA1‐dependent inner membrane fusion exacerbates mitochondrial dysfunction, bioenergetic collapse, and apoptotic signaling, thereby identifying mitochondrial dynamics as a promising therapeutic target in ischemic stroke [75].

### 4.4 BBB Disruption and NVU Injury

**FIGURE 4:**
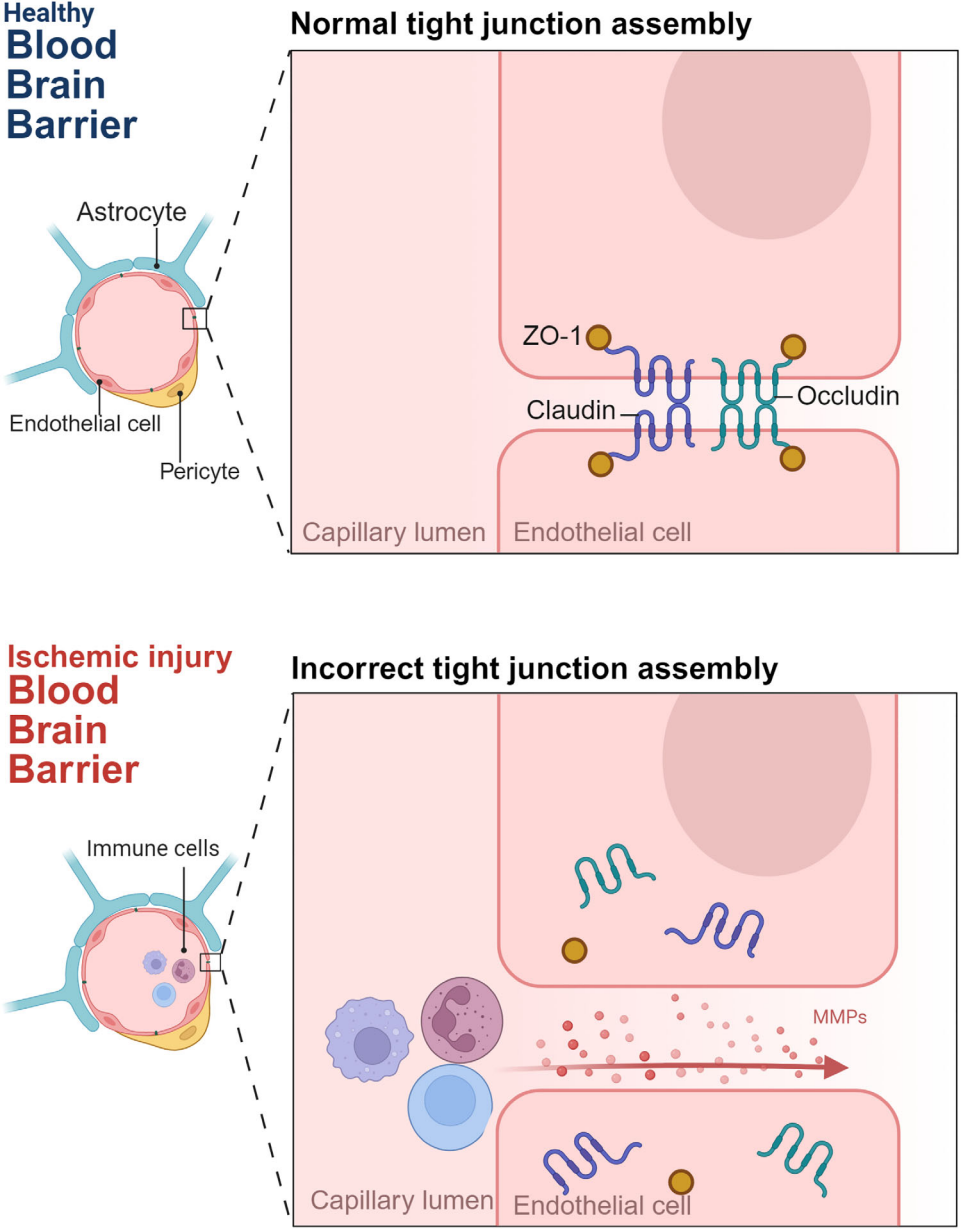
Blood–brain barrier (BBB) integrity before and after ischemic injury. This schematic compares the structural components of the healthy and compromised neurovascular unit. In the healthy state (upper panel), tight junction proteins (claudin, occludin, ZO‐1) between endothelial cells, supported by pericytes and astrocytic end‐feet, maintain a functional barrier. Following ischemic injury (lower panel), upregulation of matrix metalloproteinases (MMPs) degrades these tight junctions. This disruption leads to increased BBB permeability, allowing for immune cell infiltration and contributing to vasogenic edema.

The BBB is a critical determinant of stroke severity and outcome. Ischemia induces rapid degradation of tight junction proteins, including claudin‐5, occludin, and ZO‐1, largely mediated by matrix metalloproteinases such as MMP‐2 and MMP‐9 [76]. As schematically illustrated in Figure 4 (lower panel), disruption of tight junction assembly contributes to increased BBB permeability, facilitating vasogenic edema, extravasation of plasma proteins, and infiltration of peripheral immune cells, thereby amplifying neuroinflammation and tissue damage [77].

The concept of the NVU—comprising neurons, astrocytes, endothelial cells, pericytes, microglia, and the extracellular matrix—has reframed stroke pathophysiology as an integrated multicellular disorder rather than a neuron‐centric disease [78]. Figure 4 (upper panel) highlights the coordinated interactions among endothelial cells, pericytes, and astrocytic end‐feet that maintain BBB integrity under physiological conditions, whereas the lower panel depicts ischemia‐induced NVU disruption. Stroke‐induced NVU disintegration disrupts neurovascular coupling, impairs metabolic support, and compromises long‐term repair processes. Importantly, NVU dysfunction provides a mechanistic explanation for hemorrhagic transformation and for the frequent dissociation between angiographic recanalization and effective tissue reperfusion [79].

### 4.5 Neuroinflammation and Immune–Vascular Crosstalk

Neuroinflammation is a core driver of secondary ischemic injury and unfolds over hours to days following stroke onset. Microglia are activated within minutes, adopting context‐dependent phenotypes that range from proinflammatory to reparative [80]. Activated microglia release cytokines, including interleukin (IL)‐1β, TNF‐α, and IL‐6, as well as chemokines and ROS, thereby amplifying neuronal injury and recruiting peripheral immune cells [81].

Peripheral immune infiltration further exacerbates tissue damage. Neutrophils and monocytes traverse the activated endothelium and accumulate within the ischemic brain [82]. Neutrophil extracellular traps (NETs) obstruct microvessels, worsen BBB disruption, and promote thrombosis, providing a direct mechanistic link between inflammation and impaired reperfusion [83]. Adaptive immune responses, including T‐cell‐mediated cytotoxicity and poststroke immunosuppression, further shape clinical outcomes and susceptibility to infection [84]. These findings underscore the importance of immune–vascular crosstalk as both a driver of injury and a potential therapeutic target.

### 4.6 Regulated Cell Death Pathways: Beyond Apoptosis

Ischemic neurons die through multiple, partially overlapping regulated cell‐death programs, reflecting the diversity of molecular insults encountered across ischemic territories.

Apoptosis predominates in the penumbra and is mediated by caspase activation and BCL‐2 family signaling, allowing for a limited therapeutic window for intervention [85]. Necroptosis, driven by RIPK1/RIPK3/MLKL signaling, induces inflammatory membrane rupture and has emerged as a contributor to infarct expansion, particularly under conditions of caspase inhibition [86]. Ferroptosis—characterized by iron‐dependent lipid peroxidation and inactivation of GPX4—has gained intense interest as a druggable pathway linking oxidative stress, iron metabolism, and mitochondrial dysfunction [87, 88, 89].

Pyroptosis, mediated by inflammasome activation (e.g., NLRP3) and gasdermin D pore formation, couples cell death to robust cytokine release, thereby amplifying inflammation [90]. Autophagy dysregulation exhibits a dual role: adaptive clearance of damaged organelles versus excessive self‐digestion culminating in cell death [91]. Recognition of this cell‐death spectrum has shifted therapeutic strategies toward pathway‐specific modulation rather than indiscriminate neuroprotection.

### 4.7 Metabolic Reprogramming and Systemic Influences

Ischemic brain tissue undergoes profound metabolic reprogramming, including a shift toward anaerobic glycolysis, lactate accumulation, and altered amino acid and lipid metabolism [92]. Astrocyte–neuron metabolic coupling, including lactate shuttling and ketone utilization, has emerged as a determinant of ischemic tolerance and recovery [93]. Mitochondrial substrate flexibility may confer resilience under fluctuating oxygen and glucose availability.

Systemic metabolic states—such as diabetes, obesity, and hepatic steatosis—prime the brain for exaggerated inflammatory and oxidative responses, partly explaining interindividual variability in stroke severity and recovery [94, 95]. These observations highlight the importance of systemic metabolic health as a modifier of cerebral ischemic injury.

### 4.8 Implications for Precision Medicine

Collectively, these mechanisms underscore that cerebral infarction is not a singular disease entity but a convergence of heterogeneous injury programs. Integrating advanced imaging phenotypes with molecular signatures—such as ferroptotic activity, inflammatory burden, or endothelial injury markers—offers a rational path toward mechanism‐guided patient stratification and therapy selection [96, 97, 98, 99]. Such an approach aligns with emerging precision medicine paradigms and holds promise for improving translational success.

### 4.9 Conclusion

The mechanistic landscape of cerebral infarction has evolved from a simplistic ischemic cascade to a multidimensional network disorder encompassing vascular failure, metabolic collapse, immune dysregulation, and diverse forms of regulated cell death. These processes unfold in a highly context‐dependent manner, shaped by time, tissue compartment, systemic health, and genetic background. Crucially, this refined understanding explains the historical failure of one‐size‐fits‐all neuroprotection and provides a strong biological foundation for precision medicine. As advanced imaging, multiomics profiling, and AI‐driven analytics converge, dominant injury pathways in individual patients may soon be identified in real time, enabling targeted interventions that maximize tissue salvage and functional recovery. Mechanism‐informed therapeutics, rather than empiricism alone, are poised to redefine the future of ischemic stroke care.

## 5 Diagnostic Advances in Cerebral Infarction

Accurate and timely diagnosis constitutes the cornerstone of effective cerebral infarction management, underpinning virtually every stage of clinical decision‐making, including acute reperfusion therapy, etiological classification, prognostic stratification, and secondary prevention. Historically, stroke diagnosis relied heavily on clinical presentation and noncontrast imaging to exclude hemorrhage, with therapeutic eligibility largely governed by rigid time windows. However, this paradigm has progressively shifted over the past decade toward tissue‐based, mechanism‐oriented, and risk‐informed diagnostic frameworks.

This transformation has been catalyzed by rapid advances in multimodal neuroimaging, circulating and molecular biomarkers, high‐throughput multiomics technologies, and computational analytics, particularly AI‐driven approaches. Modern diagnostics now aim not only to confirm the presence of ischemia, but also to delineate infarct core and penumbra, characterize vascular pathology, identify stroke mechanisms, predict complications such as hemorrhagic transformation or cerebral edema, and guide individualized treatment pathways.

In this context, cerebral infarction is increasingly conceptualized as a heterogeneous, dynamic, and system‐level disorder rather than a uniform vascular event. Diagnostic tools are therefore evolving from binary classifiers toward integrated platforms that synthesize spatial, temporal, molecular, and computational information. In this section, we synthesize recent progress in cerebral infarction diagnostics, spanning neuroimaging, blood‐based and molecular biomarkers, genetic and multiomics profiling, as well as emerging point‐of‐care and AI‐enabled technologies, highlighting their collective roles in enabling precision stroke medicine [97, 100, 101, 102, 103, 104].

### 5.1 Neuroimaging‐Based Diagnostics

#### 5.1.1 Computed Tomography and Perfusion Imaging

Noncontrast computed tomography (NCCT) remains the first‐line imaging modality in the acute evaluation of suspected cerebral infarction, owing to its rapid acquisition, widespread availability, and high sensitivity for excluding intracranial hemorrhage. Although early ischemic changes on NCCT—such as subtle hypoattenuation, loss of gray–white matter differentiation, and sulcal effacement—are often difficult to detect in the hyperacute phase, their presence is associated with irreversible tissue injury and poorer outcomes.

To improve diagnostic reliability and interobserver agreement, standardized scoring systems such as the Alberta Stroke Program Early CT Score (ASPECTS) have been widely adopted. ASPECTS not only enhances the detection of early ischemic changes but also provides prognostic information and informs patient selection for reperfusion therapies [105, 106, 107].

CT angiography (CTA) has become indispensable in modern stroke workflows. Beyond its central role in identifying large‐vessel occlusions (LVOs), CTA enables assessment of extracranial and intracranial arterial anatomy, detection of tandem lesions, and evaluation of collateral circulation. Robust collateral networks, as assessed by CTA‐based collateral scoring systems, are strongly associated with slower infarct progression, reduced infarct volume, and improved response to reperfusion therapy [108, 109, 110].

CT perfusion (CTP) imaging represents a major diagnostic advance by enabling quantitative assessment of cerebral hemodynamics, including CBF, cerebral blood volume, and mean transit time. By operationally defining irreversibly injured infarct core and potentially salvageable ischemic penumbra, CTP has facilitated the extension of reperfusion therapy windows well beyond conventional time limits. Landmark trials employing CTP‐based selection have demonstrated substantial benefit of thrombectomy in late‐presenting and wake‐up stroke patients, firmly establishing tissue‐based diagnostics as a clinical standard [111, 112, 113, 114, 115].

#### 5.1.2 Magnetic Resonance Imaging

MRI is widely regarded as the gold standard for tissue‐level characterization of cerebral infarction. DWI detects cytotoxic edema within minutes of ischemia onset and offers unparalleled sensitivity for acute infarction, including small cortical or posterior circulation lesions that may be occult on CT [116, 117, 118].

The combination of DWI with fluid‐attenuated inversion recovery (FLAIR) imaging has yielded the clinically impactful DWI–FLAIR mismatch concept, which serves as a surrogate marker of stroke onset timing. This approach has enabled the safe administration of intravenous thrombolysis (IVT) in patients with unknown or unwitnessed symptom onset, thereby expanding treatment eligibility [119, 120].

Advanced MRI techniques provide further mechanistic insights. MR perfusion imaging parallels CTP in delineating penumbral tissue while avoiding ionizing radiation. Susceptibility‐weighted imaging enables detection of cerebral microbleeds, hemorrhagic transformation, and thrombus composition, informing both acute treatment and secondary prevention strategies. VWI has emerged as a particularly powerful modality for differentiating intracranial atherosclerosis, vasculitis, reversible cerebral vasoconstriction syndrome, and arterial dissection—conditions that are often indistinguishable using conventional luminal imaging alone [21, 22, 23, 24, 105, 121, 122, 123, 124, 125, 126].

### 5.2 Circulating and Molecular Biomarkers

Despite extensive investigation, no single circulating biomarker has yet achieved sufficient sensitivity and specificity to independently diagnose acute ischemic stroke in routine clinical practice. Nonetheless, a growing body of evidence supports the utility of several biomarkers as adjunctive diagnostic, prognostic, and stratification tools.

Glial fibrillary acidic protein (GFAP), a marker of astroglial injury, has demonstrated particular promise in distinguishing ischemic stroke from intracerebral hemorrhage in the hyperacute setting, potentially aiding prehospital triage and early therapeutic decisions [127, 128, 129]. Neurofilament light chain (NfL), reflecting axonal injury, correlates robustly with infarct volume, stroke severity, and long‐term functional outcome, and may serve as a dynamic marker of ongoing neuroaxonal damage [130, 131, 132, 133].

Additional candidates include markers of neuronal injury (tau protein), astrocytic activation (S100B), endothelial dysfunction, oxidative stress, and systemic inflammation, such as C‐reactive protein and IL‐6 [134, 135, 136]. Importantly, recent studies suggest that multimarker panels, integrating biomarkers from distinct pathophysiological domains, outperform individual markers, reinforcing the concept that cerebral infarction diagnosis and prognostication require a multidimensional approach [137, 138, 139].

### 5.3 Genetic and Multiomics Approaches

Genetic studies have substantially advanced understanding of stroke susceptibility and heterogeneity. GWAS have identified numerous loci associated with overall stroke risk as well as subtype‐specific predispositions, shedding light on pathways related to lipid metabolism, coagulation, inflammation, and vascular integrity [33, 140].

Beyond genomics, high‐throughput transcriptomics, proteomics, metabolomics, and lipidomics are increasingly applied to blood and cerebrospinal fluid samples. These approaches have revealed that ischemic stroke induces profound, time‐dependent alterations in immune signaling, metabolic pathways, and coagulation cascades, some of which exhibit distinct signatures across stroke subtypes [141, 142, 143, 144, 145].

Integrative multiomics models, which combine data across molecular layers, have demonstrated potential for differentiating cardioembolic from large‐artery atherosclerotic stroke, predicting early neurological deterioration, and forecasting recovery trajectories. Although currently limited by cost, technical complexity, and lack of standardized analytical pipelines, multiomics diagnostics represent a conceptual foundation for future precision stroke medicine [91, 146, 147].

### 5.4 AI and Computational Diagnostics

AI and ML have rapidly permeated stroke diagnostics, particularly within neuroimaging. AI algorithms can automatically detect LVOs on CTA, quantify infarct core and penumbra on perfusion imaging, and identify subtle early ischemic changes on NCCT with accuracy comparable to expert neuroradiologists, while dramatically reducing interpretation time [148, 149, 150, 151, 152].

Radiomics approaches extract high‐dimensional quantitative features from imaging data that are imperceptible to human observers. When combined with ML classifiers, radiomics models can predict hemorrhagic transformation, collateral status, infarct growth, and functional outcome. These tools offer a pathway toward objective, reproducible, and scalable diagnostic assessments [153, 154, 155].

Importantly, AI‐driven decision‐support systems enhance diagnostic consistency, mitigate interobserver variability, and improve access to expert‐level interpretation in resource‐limited settings. However, challenges related to data bias, generalizability, and regulatory oversight remain key barriers to widespread clinical adoption.

### 5.5 Point‐of‐Care and Prehospital Diagnostics

Efforts to decentralize and accelerate stroke diagnosis have intensified in recent years. Portable CT and low‐field MRI systems are being evaluated for use in ambulances, mobile stroke units, and rural hospitals, with the goal of reducing door‐to‐needle and door‐to‐groin times [156, 157, 158].

Concurrently, point‐of‐care biosensors capable of rapidly detecting stroke‐associated biomarkers are under active development. When integrated with telemedicine networks, these tools enable early diagnosis, prehospital triage, and direct routing of patients to thrombectomy‐capable centers. Such innovations hold particular promise for addressing global disparities in stroke care and improving outcomes in underserved regions [159, 160].

### 5.6 Conclusion

Diagnostic advances in cerebral infarction have undergone a profound transformation, evolving from exclusion‐based imaging and symptom‐driven assessment toward a multidimensional, precision‐oriented framework. Contemporary diagnostics integrate advanced neuroimaging, circulating and molecular biomarkers, genetic and multiomics profiling, and AI‐driven computational analytics to characterize tissue viability, elucidate stroke mechanisms, and inform individualized therapeutic strategies.

While challenges remain—particularly with respect to cost, accessibility, standardization, and clinical validation—the convergence of these technologies is steadily eroding the limitations of traditional time‐based paradigms. As diagnostic tools continue to mature and become more widely implemented, they are poised to redefine stroke care by enabling earlier intervention, improved patient selection, and ultimately better functional outcomes in cerebral infarction.

## 6 Management Strategies of Cerebral Infarction

Management of cerebral infarction has entered an era characterized by rapid technological advancement and increasing biological sophistication. While early paradigms emphasized rigid time windows and uniform treatment algorithms, contemporary strategies increasingly rely on tissue viability, vascular anatomy, and patient‐specific biological profiles. Acute reperfusion remains the only proven disease‐modifying therapy, yet its benefits are constrained by eligibility criteria, incomplete microvascular reperfusion, and reperfusion injury. Meanwhile, secondary prevention has shifted toward etiology‐driven and pharmacogenomically informed strategies. Parallel progress in neuroprotection, immunomodulation, regenerative medicine, and neurorehabilitation reflects a broader recognition that recovery after cerebral infarction is governed by dynamic interactions within the NVU and systemic immune–metabolic networks. This section synthesizes current evidence across the continuum of care, highlighting both established therapies and emerging interventions that may collectively redefine the therapeutic landscape.

### 6.1 Acute Phase Management and Reperfusion Therapies

#### 6.1.1 IVT: Optimization and Extension of Therapeutic Windows

IVT remains the most accessible reperfusion strategy worldwide. Alteplase (rt‐PA) administered within 4.5 h has long been standard; however, tenecteplase has gained momentum due to its pharmacological advantages, including greater fibrin specificity and longer half‐life. Multiple randomized controlled trials (RCTs) and meta‐analyses demonstrate noninferiority or superiority of tenecteplase in achieving early reperfusion, especially prior to mechanical thrombectomy (MT) in LVO [161, 162, 163, 164].

Imaging‐guided thrombolysis has further expanded treatment eligibility. Trials such as WAKE‐UP and EXTEND demonstrated that MRI diffusion–FLAIR mismatch or CTP‐based penumbral selection can identify patients who benefit from thrombolysis beyond conventional time windows or in wake‐up strokes [165, 166]. These findings reinforce the paradigm shift from clock‐based to tissue‐based decision‐making.

Nevertheless, hemorrhagic transformation remains a major limiting factor. Predictive models integrating age, baseline NIHSS, glucose, infarct core volume, and radiomic features are increasingly explored using ML approaches to refine patient selection and personalize risk assessment [167, 168].

#### 6.1.2 MT: Technological Refinement and Systems of Care

MT has transformed outcomes for LVO stroke, with robust benefit demonstrated up to 24 h in imaging‐selected patients [169, 170]. Technological innovations—including large‐bore aspiration catheters, improved stent retrievers, and combined techniques—have significantly increased rates of first‐pass reperfusion, now recognized as a strong predictor of favorable functional outcome [171].

Despite high rates of macrovascular recanalization, incomplete microvascular reperfusion persists in many patients, contributing to infarct expansion and poor outcomes. Mechanisms include distal embolization, endothelial swelling, microthrombosis, pericyte‐mediated capillary constriction, and inflammatory vascular plugging [172, 173]. These insights have stimulated interest in adjunctive therapies targeting microcirculatory dysfunction.

#### 6.1.3 Bridging Therapy Versus Direct Thrombectomy

Whether IV thrombolysis should precede MT remains debated. Several RCTs comparing direct MT with bridging therapy have yielded heterogeneous results, with some Asian trials suggesting noninferiority of direct MT, while others indicate potential benefit of combined therapy [174, 175, 176]. Differences in clot composition, collateral status, and stroke etiology likely modulate therapeutic response, underscoring the need for individualized treatment algorithms.

### 6.2 Secondary Prevention and Long‐Term Vascular Protection

#### 6.2.1 Antithrombotic Strategies and Pharmacogenomics

For noncardioembolic stroke, short‐term dual antiplatelet therapy (DAPT) with aspirin and clopidogrel significantly reduces early recurrence in minor stroke or high‐risk TIA [177, 178]. Long‐term DAPT, however, confers increased hemorrhagic risk without added benefit, emphasizing the importance of temporal precision in therapy.

In cardioembolic stroke, particularly atrial fibrillation‐related infarction, direct oral anticoagulants (DOACs) have become first‐line therapy due to lower intracranial bleeding risk and fewer drug interactions compared with warfarin [178, 179]. Ongoing trials continue to refine optimal timing of anticoagulation initiation after acute stroke to balance ischemic and hemorrhagic risks.

Pharmacogenomic testing, especially CYP2C19 loss‐of‐function allele screening, identifies patients with reduced clopidogrel responsiveness who may benefit from alternative agents such as ticagrelor, illustrating a practical implementation of precision medicine [180].

#### 6.2.2 Lipid, Blood Pressure, and Metabolic Control

Aggressive lipid lowering is central to secondary prevention. Evidence supports intensive statin therapy, and recent data suggest that achieving very low LDL‐C targets further reduces recurrence, particularly in atherosclerotic stroke [181, 182]. PCSK9 inhibitors are increasingly considered in high‐risk patients with inadequate statin response.

Blood pressure management remains the most effective population‐level preventive strategy, yet individualized targets may be necessary in patients with severe intracranial stenosis or impaired cerebral autoregulation to avoid hypoperfusion.

#### 6.2.3 Etiology‐Specific Interventions

Mechanism‐driven prevention includes carotid endarterectomy or stenting for symptomatic carotid stenosis, patent foramen ovale (PFO) closure in selected cryptogenic strokes, and targeted management of atrial cardiopathy [183, 184]. These interventions highlight the importance of precise etiological diagnosis.

### 6.3 Neuroprotection and Immunomodulation

#### 6.3.1 Lessons from Failed Neuroprotection Trials

Despite extensive preclinical success, translation of neuroprotection into clinical benefit has largely failed. Key reasons include lack of comorbidity in animal models, failure to combine with reperfusion, inadequate CNS drug delivery, and patient heterogeneity in clinical trials [73]. These lessons have reshaped contemporary strategies toward combination therapy and biomarker‐guided patient stratification.

#### 6.3.2 Targeting Ferroptosis, Mitochondrial Dysfunction, and Oxidative Stress

Ferroptosis, characterized by iron‐dependent lipid peroxidation and GPX4 inactivation, has emerged as a major contributor to ischemic neuronal death. Inhibitors of lipid peroxidation and iron chelators reduce infarct size in preclinical models, positioning ferroptosis as a promising therapeutic target [56, 72, 185, 186].

Mitochondrial dysfunction is central to energy failure and apoptosis. Therapeutic strategies targeting mitochondrial permeability transition, ROS production, and mitophagy are under active investigation [187].

#### 6.3.3 Neuroinflammation and Immune Modulation

Poststroke inflammation involves microglial activation, peripheral immune cell infiltration, cytokine cascades, and NET formation. Therapeutic strategies include IL‐1 receptor antagonists, NLRP3 inflammasome inhibitors, and agents targeting neutrophil–endothelial interactions [188, 189, 190, 191]. While early‐phase trials suggest biological plausibility, large‐scale efficacy data remain limited.

#### 6.3.4 Combination Therapy with Reperfusion

Neuroprotection is increasingly conceptualized as adjunctive to reperfusion rather than independent therapy. Combining thrombectomy with anti‐inflammatory or antioxidative agents may mitigate reperfusion injury, reduce microvascular no‐reflow, and improve tissue salvage [192].

Table 2 summarizes the key acute management strategies, secondary prevention approaches, and neuroprotective interventions described in Sections 6.1–6.3.

**TABLE 2:** Acute management and secondary prevention strategies in ischemic stroke.

Category	Intervention	Key points
Acute phase management	IV thrombolysis [161, 162, 163, 164, 165, 166, 167, 168]	Alteplase ≤4.5 h; tenecteplase superior pharmacology; imaging‐guided selection extends window; hemorrhage risk prediction
	Mechanical thrombectomy [169, 170, 171, 172, 173]	First‐pass recanalization improves outcome; microvascular no‐reflow limits recovery; adjunct therapies under study
	Bridging vs. direct MT [174, 175, 176]	Mixed RCT results; outcome influenced by clot, collaterals, etiology
Secondary prevention	Antithrombotics and pharmacogenomics [177, 178, 179, 180]	Short‐term DAPT for minor stroke/TIA; DOACs for AF; CYP2C19 genotyping guides therapy
	Lipid/BP/metabolic control [181, 182]	Intensive statins; PCSK9 inhibitors for high‐risk; individualized BP targets
	Etiology‐specific interventions [183, 184]	Carotid revascularization; PFO closure; atrial cardiopathy management
Neuroprotection and immunomodulation	Lessons from trials [73]	Translational failures highlight need for reperfusion combination and biomarker‐guided therapy
	Ferroptosis and mitochondria [55, 72, 185, 186, 187]	Target lipid peroxidation, iron overload, ROS, mitophagy
	Neuroinflammation [188, 189, 190, 191]	IL‐1R, NLRP3 inhibitors; neutrophil–endothelial modulation; early‐phase evidence
	Combination with reperfusion [192]	Adjunctive therapy may reduce reperfusion injury, improve tissue salvage

### 6.4 Regenerative Medicine and Neural Repair

#### 6.4.1 Stem Cell and Exosome‐Based Therapies

Stem cell therapies primarily exert paracrine effects, promoting angiogenesis, neurogenesis, and immune modulation rather than direct neuronal replacement. Mesenchymal stem cells and neural progenitor cells have demonstrated safety and modest functional benefit in early‐phase trials [193, 194, 195, 196].

Exosome‐based therapies may overcome limitations of cell survival and immunogenicity, delivering microRNAs and trophic factors that modulate poststroke repair pathways [197].

#### 6.4.2 Growth Factors and Endogenous Plasticity

Growth factors such as G‐CSF and BDNF analogues aim to enhance endogenous repair mechanisms. Translation has been limited by systemic adverse effects and narrow therapeutic windows, but combinatorial approaches with rehabilitation may enhance efficacy [198].

#### 6.4.3 Neuromodulation and Brain–Computer Interfaces

Noninvasive neuromodulation techniques, including TMS and tDCS, can modulate cortical excitability and enhance training‐induced plasticity. Brain–computer interfaces offer closed‐loop feedback systems that facilitate motor recovery by directly linking neural signals to assistive devices [199, 200, 201].

### 6.5 Rehabilitation, Digital Health, and Long‐Term Outcomes

Recovery after stroke depends on activity‐dependent plasticity. Early mobilization, high‐intensity task‐oriented training, robotic‐assisted therapy, and virtual reality platforms allow delivery of high‐dose, individualized rehabilitation [202].

Wearable sensors and digital biomarkers enable continuous monitoring of recovery trajectories, allowing adaptive therapy and improved adherence. Tele‐rehabilitation expands access, particularly in underserved regions [203, 204].

Management of cognitive impairment, depression, fatigue, and sleep disorders is essential for long‐term quality of life and reintegration into society [205].

### 6.6 Conclusion

Therapeutic strategies for cerebral infarction are transitioning from singular focus on vessel recanalization toward an integrated, precision‐oriented continuum of care encompassing reperfusion, neuroprotection, immune modulation, vascular repair, and neurorehabilitation. While MT and thrombolysis remain indispensable, their limitations—particularly microvascular dysfunction and reperfusion injury—necessitate adjunctive biological therapies. Advances in molecular neuroscience have identified novel targets such as ferroptosis and inflammasome signaling, while regenerative medicine and neuromodulation offer promising avenues for functional restoration. Ultimately, integration of multimodal imaging, molecular profiling, and AI is expected to enable individualized therapeutic pathways, optimizing both survival and long‐term neurological recovery. Achieving this vision will require coordinated translational research, innovative trial designs, and equitable healthcare implementation.

## 7 Emerging Technologies and Precision Medicine in Cerebral Infarction

The concept of precision medicine in cerebral infarction seeks to transcend traditional one‐size‐fits‐all models of stroke care by tailoring prediction, diagnosis, and treatment strategies to an individual's unique biological, clinical, and environmental profile. Historically, stroke therapeutics have been driven by broad clinical indicators (e.g., time since onset) and population‐level risk factors such as hypertension or diabetes. However, these approaches inadequately capture the substantial heterogeneity of ischemic stroke mechanisms, outcomes, and therapeutic responses observed across patients. Personalized strategies must integrate multiple data streams—including genomics, transcriptomics, proteomics, metabolomics, clinical imaging, and machine‐learned insights—to define mechanistic endophenotypes that better predict risk, optimize intervention selection, and improve functional outcomes across the acute and chronic phases of stroke [156, 206, 207].

Precision medicine in stroke is now being accelerated by three interconnected technological domains: AI and ML, multiomics integration, and point‐of‐care biomarker discovery and deployment. Collectively, these technologies hold the promise of enabling ultra‐early risk stratification, dynamic disease monitoring, and individualized therapeutic decisions that align with the biological reality of each patient's infarct progression [159, 208, 209].

### 7.1 AI‐Assisted Risk Prediction and Clinical Decision Support

AI has emerged as a cornerstone of precision medicine for cerebral infarction by enabling the automated analysis of high‐dimensional datasets derived from clinical records, imaging modalities, and other biomarkers. ML models can identify complex patterns within these datasets that evade human interpretation, allowing for individualized outcome prediction and optimized clinical decision support [208, 209, 210, 211].

Large‐scale ML algorithms trained on multimodal data (clinical, demographic, laboratory, and imaging variables) can forecast functional outcomes, therapeutic responses, and complication risks for individual patients. For example, predictive models incorporating diffusion MRI features and clinical variables have achieved high accuracy for 3‐month outcome prediction, outperforming traditional scoring approaches [212]. Similarly, AI models designed to assess angiographic reperfusion and guide endovascular treatment decision‐making have demonstrated robust correlations with expert assessments of reperfusion success.

Beyond outcome prediction, AI is being applied to automate the detection of LVOs, quantify infarct core and penumbral volumes on perfusion imaging, and distinguish stroke subtypes from imaging data—a foundational step for personalized therapy allocation. Real‐world implementations of AI‐based tools have shown reduced triage times, while still requiring clinician oversight to contextualize machine outputs within clinical nuance [213].

Key challenges remain in AI deployment—including algorithm transparency, data privacy, and bias mitigation—but advances in explainable AI and federated learning are helping to address these concerns. Federated AI frameworks enable model training across institutional silos without direct data sharing, preserving patient confidentiality while expanding model generalizability across diverse populations [214].

### 7.2 Multiomics Integration for Subtype Classification and Mechanism Elucidation

Multiomics approaches integrate genomic, transcriptomic, proteomic, and metabolomic data to uncover the molecular architecture underlying cerebral infarction and its diverse subtypes. High‐throughput sequencing and mass spectrometry technologies have made it feasible to generate comprehensive molecular profiles at scale, revealing disease pathways that contribute to stroke risk, progression, and recovery trajectories [215, 216, 217].

Recent integrative analyses have identified novel genetic loci associated with ischemic stroke, implicating genes related to inflammation, vascular function, and metabolism, and providing a foundation for genotype‐informed precision strategies [216, 218, 219]. In parallel, genomic and biomarker characterization efforts have delineated molecular signatures capable of stratifying patients beyond traditional clinical classifications, facilitating improved risk prediction and mechanistic understanding [218, 220].

Multiomics models are also being leveraged for etiology classification, circumventing the limitations of purely symptom‐based or imaging‐based systems. By identifying patterns of DNA methylation, mRNA, and noncoding RNA expression, these strategies are yielding predictive frameworks that can discriminate between underlying stroke mechanisms and potentially reveal new therapeutic targets [219]. Integration of omics layers through advanced computational models such as knowledge graphs and network inference further enhances the discovery of molecular interactions that may serve as therapeutic levers [221].

### 7.3 ML Models for Treatment Selection and Response Prediction

ML models are extending precision within stroke care by predicting individual treatment responses, enabling clinicians to optimize reperfusion strategies and secondary prevention regimens on a per‐patient basis. These models leverage personalized clinical variables, imaging biomarkers, and molecular profiles to estimate the likelihood of benefit or harm from interventions such as thrombolysis or anticoagulation [222, 223, 224].

For instance, outcome prediction algorithms incorporating MRI and clinical features have been used to tailor decisions about endovascular therapy, while ML frameworks assessing risk factors and treatment responses in atrial fibrillation patients enhance primary and secondary stroke prevention strategies through refined anticoagulant selection [225]. Increasingly, ML frameworks are integrating pharmacogenomic data—such as CYP2C19 metabolizer status—to guide antiplatelet therapy choices [226].

### 7.4 Circulating and Molecular Biomarkers for Personalized Diagnostics

Blood‐based biomarkers are essential for realizing precision diagnostics that can be portable, rapid, and mechanistically informative. Beyond classical proteins like GFAP, which aid in distinguishing ischemic from hemorrhagic stroke, novel biomarker panels incorporating NfL, cardiac peptides, and inflammatory signals are emerging as tools for subtype classification, prognosis, and therapeutic monitoring [227, 228].

Metabolomics studies have identified combinations of amino acids, lipids, and other small molecules that show high discriminative power for acute ischemic stroke and may differentiate etiologies or indicate risk of complications [229, 230, 231]. Integration of biomarker data with clinical and imaging features through ML algorithms creates composite risk profiles that can inform real‐time clinical decisions, including prehospital triage and treatment prioritization [232].

### 7.5 Point‐of‐Care and Rapid Diagnostic Technologies

Point‐of‐care devices—such as portable imaging systems, miniaturized biosensors, and NIRS‐based oxygenation monitors—are poised to transform early diagnosis and triage by delivering high‐fidelity biological signals closer to the patient [31]. AI‐enhanced near‐infrared spectroscopic patches have demonstrated the capacity to differentiate ischemic stroke patients from controls with high accuracy in preliminary studies, suggesting a path toward real‐time physiological monitoring in emergency settings.

### 7.6 Ethical, Equity, and Implementation Considerations

While the technological foundation for precision stroke medicine is rapidly advancing, several implementation challenges remain. These include addressing data privacy and algorithmic bias in AI models, ensuring equitable access to omics‐informed diagnostics in diverse healthcare systems and validating biomarkers and predictive models across heterogeneous populations.

### 7.7 Conclusion

The integration of AI‐assisted analytics, multiomics profiling, biomarker discovery, and point‐of‐care diagnostics is ushering in a transformative era for cerebral infarction care. By aligning mechanistic insights with individual patient biology, precision medicine promises more accurate risk stratification, enhanced diagnostic specificity, personalized treatment allocation, and ultimately improved outcomes across the acute and chronic phases of stroke. Continued investment in diverse data generation, computational innovation, and equitable clinical implementation will be essential to translate these advances from research proof‐of‐principle into everyday practice.

## 8 Prospects

Cerebral infarction remains a formidable global health challenge, but the field is in the midst of a transformative era. The journey from understanding its macroscopic epidemiology to deciphering the subcellular intricacies of ferroptosis and pyroptosis has been remarkable.

The clinical paradigm is shifting from a simplistic, time‐locked approach to a more sophisticated, tissue‐ and mechanism‐based model. The convergence of advanced neuroimaging, high‐throughput omics, and AI is forging a path toward true precision medicine, where risk prediction, diagnosis, and treatment will be tailored to the individual's unique pathophysiology.

While significant challenges in translation and implementation persist, the integration of these technological advancements with a deepened biological understanding holds immense translational potential. The coming decade promises to usher in a new age where the burden of cerebral infarction can be systematically reduced through earlier, smarter, and more personalized interventions, ultimately improving survival and functional outcomes for millions worldwide.

## Author Contributions

Meibiao Zhang, Jianxun Hou, and Jia Kuang participated in writing. Lei Hao and Zhao Yang participated in reviewing and editing. All authors have read and approved the final manuscript.

## Funding

This study was supported by Chongqing Science and Health Joint Medical Research Project (2025ZDXM003).

## Ethics Statement

The authors have nothing to report.

## Conflicts of Interest

The authors declare no conflicts of interest.

## Data Availability

The authors have nothing to report.
